# How to use the Kaiser score as a clinical decision rule for diagnosis in multiparametric breast MRI: a pictorial essay

**DOI:** 10.1007/s13244-018-0611-8

**Published:** 2018-04-03

**Authors:** Matthias Dietzel, Pascal A. T. Baltzer

**Affiliations:** 1 Department of Radiology, University Hospital Erlangen-Nürnberg, Maximiliansplatz 1, 91054 Erlangen, Germany; 2 Department of Biomedical Imaging and Image-Guided Therapy, Division of Molecular and Gender Imaging, Medical University of Vienna, Waehringer-Guertel, 18-20 Vienna, Austria

**Keywords:** Breast MRI, Clinical decision-making, Breast cancer, Patient management

## Abstract

**Abstract:**

Due to its superior sensitivity, breast MRI (bMRI) has been established as an important additional diagnostic tool in the breast clinic and is used for screening in patients with an elevated risk for breast cancer. Breast MRI, however, is a complex tool, providing multiple images containing several contrasts. Thus, reading bMRI requires a structured approach. A lack of structure will increase the rate of false-positive findings and sacrifice most of the advantages of bMRI as additional work-up will be required. While the BI-RADS (Breast Imaging Reporting And Data System) lexicon is a major step toward standardised and structured reporting, it does not provide a clinical decision rule with which to guide diagnostic decisions. Such a clinical decision rule, however, is provided by the Kaiser score, which combines five independent diagnostic BI-RADS lexicon criteria (margins, SI-time curve type, internal enhancement and presence of oedema) in an intuitive flowchart. The resulting score provides probabilities of malignancy that can be used for evidence-based decision-making in the breast clinic. Notably, considerable benefits have been demonstrated for radiologists with initial and intermediate experience in bMRI. This pictorial essay is a practical guide to the application of the Kaiser score in the interpretation of breast MRI examinations.

****Teaching Points**:**

*• bMRI requires standardisation of patient-management, protocols, and reading set-up.*

*• Reading bMRI includes the assessment of breast parenchyma, associated findings, and lesions.*

*• Diagnostic decisions should be made according to evidence-based clinical decision rules.*

*• The evidence-based Kaiser score is applicable independent of bMRI protocol and scanner.*

*• The Kaiser score provides high diagnostic accuracy with low inter-observer variability.*

## Background

Clinical breast MRI (bMRI) was pioneered by two European radiologists, Sylvia Heywang-Köbrunner and Werner Alois Kaiser, in the mid 1980s [1, 2]. Quickly, it became evident that this method provided the highest sensitivity for the detection of breast cancer and bMRI has been established as a major second-line imaging tool [3–8]. Although there are ongoing discussions about the optimal clinical use of this method, typical bMRI indications have been established [4, 5, 9]. Apart from high-risk screening, these indications could be simplified as: bMRI is always used if clinical and conventional radiological findings cannot fully resolve the diagnostic task of confirming or excluding breast cancer [10].

Solving diagnostic problems with breast MRI, however, is a complex task, as the sensitivity to detect potential cancers (= enhancements) is very high, but the characterisation of MRI-detected lesions requires simultaneous interpretation of multiple images that contain several contrasts [11]. Without a structured interpretation approach, the rate of false-positive findings will increase, which will sacrifice most of bMRI's advantages as additional workup is necessary. Yet, if quality management criteria are fulfilled and standardised interpretation criteria are applied, high accuracy with excellent sensitivity and specificity levels have been confirmed [12–15]. The first basic requisite for accurate diagnoses is examinations of high quality. Without robust image quality, all subsequent diagnostic steps are compromised. The second necessity is standardised reading conditions that consider the multiparametric nature of bMRI. Finally, translating imaging findings into standardised care with minimal inter-reader variation is improved by clinical decision rules. These are defined as an algorithmic combination of several criteria that lead to a diagnostic category (see cebm.net). In this respect, BI-RADS is *not* a clinical decision rule, but rather a lexicon that provides a common language for lesion description [16]. We developed such a clinical decision rule that was previously referred to as the *Tree* flowchart [16–18] and that will, from now on, be named the “Kaiser score” after the bMRI pioneer Werner A. Kaiser who set up the framework for its development.

This pictorial essay is a practical guide to the application of the Kaiser score in interpreting breast MRI examinations.

## Robust imaging

**Fig. 1:**
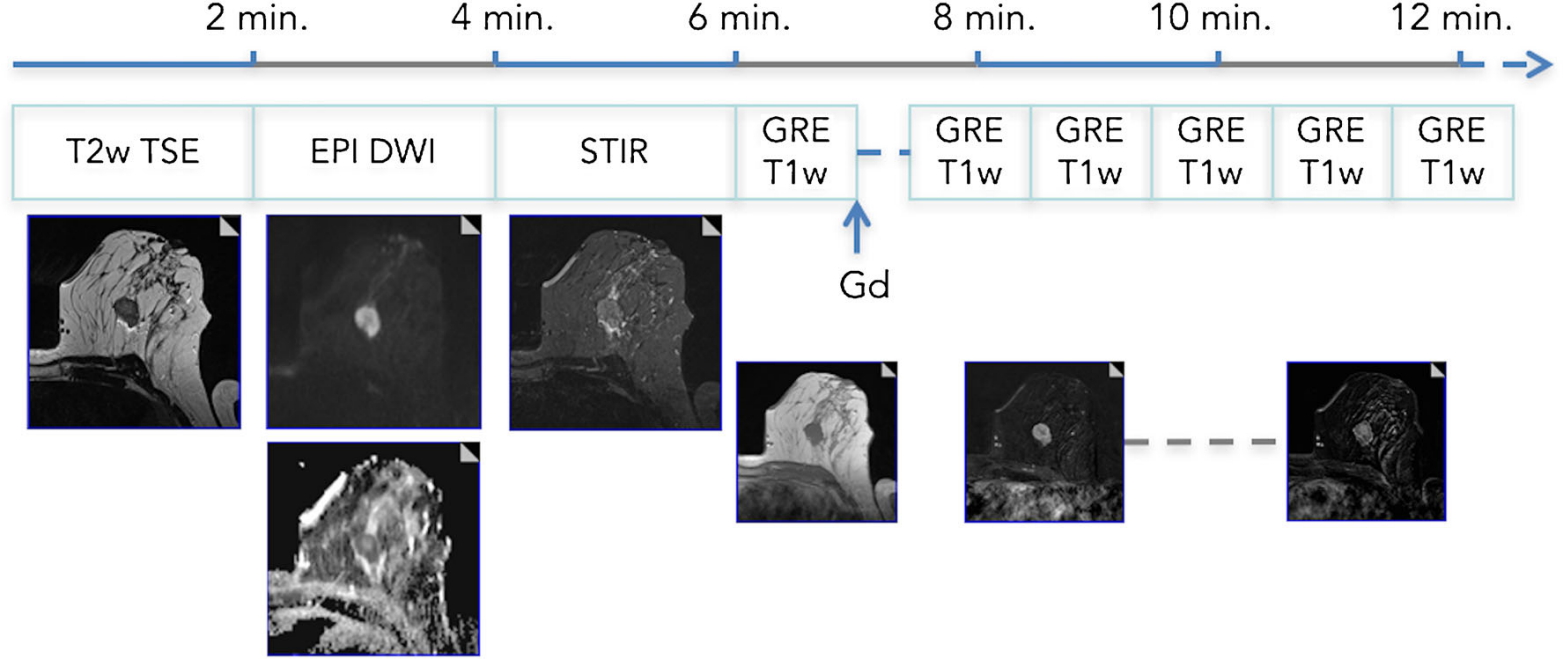
Multiparametric bMRI protocol. A full triparametric bMRI protocol can be acquired in 15 min of magnet time. Patient preparation, including venous catheter placement, should be done outside the scanner room. The (recommended) axial protocol starts with precontrast T2w-TSE, DWI, and STIR (optional) sequences. Here, a mass lesion in a 47-year-old patient with perifocal oedema and central necrosis is easily depicted. Dynamic scanning should include 4–5 min of post-contrast acquisition. The example demonstrates a type III curve, and the resulting Kaiser score is 8 (see Fig. 6), highly suggestive of malignancy. Histopathology revealed an invasive ductal cancer G3

A prerequisite for accurate diagnosis is robust imaging. Robustness in this respect refers to both patient preparation and stable image quality in adherence with predefined standards. We strongly recommend maintaining an established protocol, which should not be varied, even if, e.g., a new and promising sequence is promoted by colleagues or vendors. As opposed to other imaging, such as brain or liver, the breast lacks an inherent anatomical reference frame. Lesion appearance is thus of utmost importance and readers should familiarise themselves with the specific lesion contrast and appearance profiles of different breast pathologies as imaged by their protocol. Even when the radiologist has years of experience, a comparison with prior studies would be simpler if only one protocol was followed. This imaging protocol should be as short as possible and only include diagnostically relevant sequences (Fig. 1). A modern bMRI protocol should be triparametric and include T2-weighted, diffusion-weighted imaging (DWI) and dynamic contrast-enhanced T1-weighted spoiled gradient echo sequences. The latter is essential and a protocol without dynamic contrast-enhanced scans is not officially acknowledged as a diagnostic examination [4, 5, 9, 12]. Key aspects in image acquisition are as follows:

- *A fat saturation* might be added to T1w and/or T2w scans according to personal preferences and is mandatory in DWI. Results of spectral fat saturation strongly depend on breast tissue composition (worse in fatty breasts) and B_0_ field homogeneity (lower in higher field strengths and older MRI systems). A very attractive alternative to these fat saturation techniques for T1w and T2w imaging is the Dixon method, which is largely independent of B_0_ field homogeneity.
- Generally, the in-plane spatial resolution is much more important to the overall image impression than slice thickness. Thus, investing in non-isotropic voxels with relatively high slice thicknesses to ensure a sufficient signal-to-noise ratio (SNR) is recommended. The slice thickness should range around 2 mm, but not exceed 3 mm to avoid partial volume effects.
- *Contrast media* should be administered at a standardised dosage of 0.1 mmol/kg/body weight. Dynamic measurements should not exceed 4-5 min post injection as later scans add only little diagnostic information. The injection delay should be set in a way that ensures adequate contrast enhancement in the first scan, defined as contrast enhancement not only in arterial vessels, but also all enhancing lesions. Temporal resolution should be below 2 min.
- Basic *diffusion-weighted imaging (DWI)* should be performed in an axial orientation using two b-values consisting of one low (b 0-50 s/mm^2^) and one high value (around 800 s/mm^2^). The b0 value requires a shorter acquisition time as no diffusion-sensitising gradient is used, whereas b50 reduces perfusion effects and decreases the vessel contribution to ADC maps. SPAIR fat saturation is recommended. Spatial resolution should be anisotropic (higher in-plane resolution) and as high as SNR constraints allow.

In addition to the economic aspects, longer examinations will decrease patient compliance and result in motion artefacts that compromise diagnostic image quality. The importance of a skilled technician cannot be overemphasised. Empathic patient management is probably the most important factor in the overall quality of the examination.

## Image interpretation

**Fig. 2:**
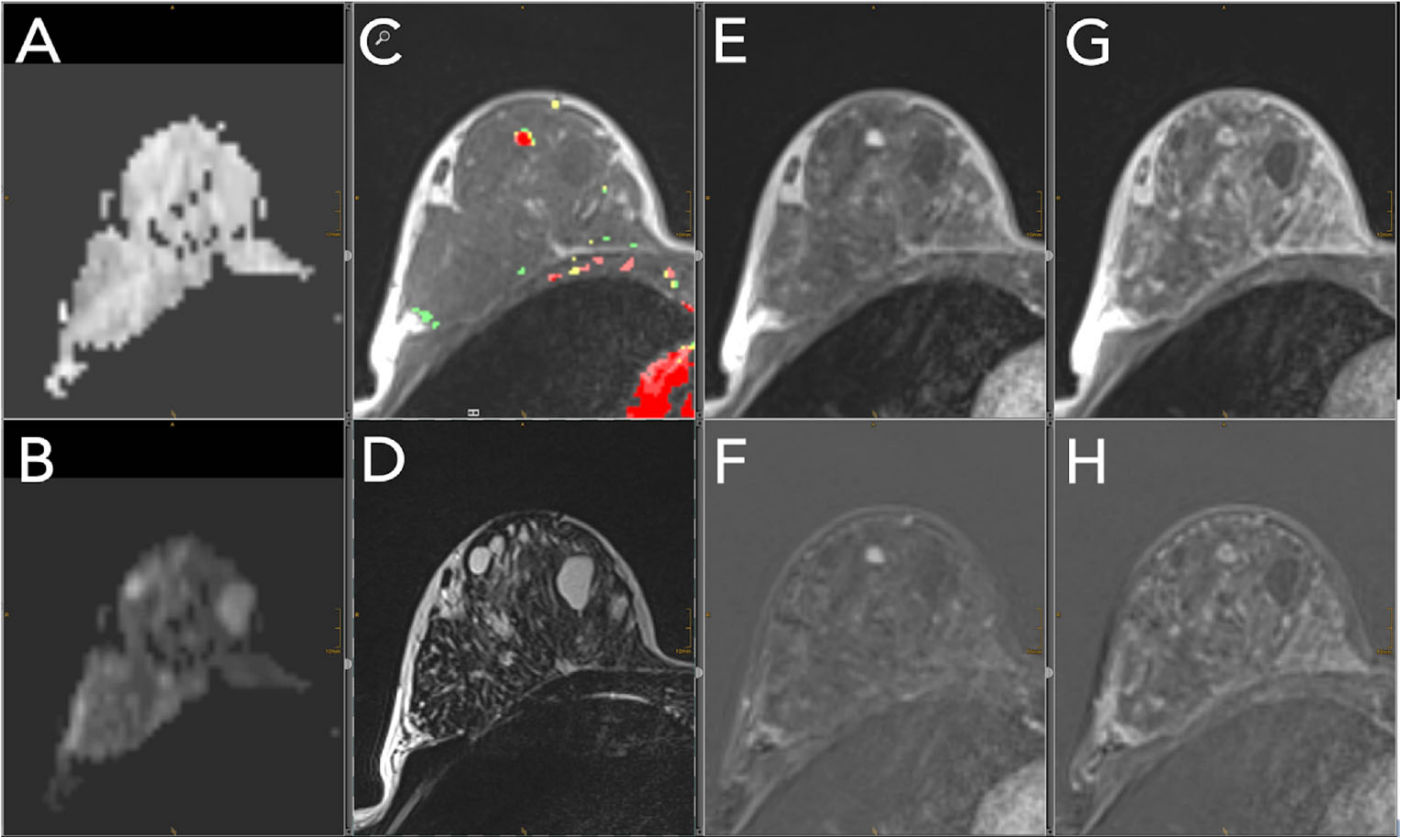
Standardised hanging protocol/reading layout for a two-screen solution. Standardised hanging protocols decrease the reading time and increase diagnostic confidence while reducing potential sources of errors. All series should be linked, allowing simultaneous scrolling and zooming. This example shows a 44-year-old high-risk patient. A: ADC map; B: high b-value DWI image; C: pre-contrast T1w (here with superimposed colour-coded enhancement map indicating dominant wash-out, coded in red); D: T2w-TSE; E/F: early contrast-enhanced scan with corresponding subtraction; G/H: delayed contrast-enhanced scan with corresponding subtraction within the dynamic series (E–H); the same windows and centre setting should be used. This step should be performed for subtracted and non-subtracted images separately. An incidental lesion of 5 mm in size is present in the right breast, showing homogeneous enhancement (E, F), followed by early and central wash-out (C, G, H). Intermediate T2w-signal [D, no restricted diffusivity (A, B)]. The lesion corresponds to a Kaiser score of 4 (see Fig. 6); MRI-guided biopsy revealed a benign adenosis B2

To accelerate the bMRI reading workflow and reduce potential sources of error, a standardised hanging protocol or layout is recommended. The relevant series should be linked, and a standardised window/centre-setting should be applied. This allows the simultaneous and true multiparametric interpretation of all relevant contrasts with various levels of magnification at a glance. An example of a hanging protocol is given in Fig. 2. Reading bMRI should adhere to the syntax and vocabulary of the MRI BI-RADS lexicon. It provides a standardised language for reporting and thus ensures reports that are understood between departments and institutions. Before lesion assessment, the amount of fibroglandular tissue and background parenchymal enhancement is assessed, as detailed elsewhere [19].

## Lesion identification and description

A bMRI lesion is defined as any enhancing structure that cannot be attributed to normal anatomy, including background parenchymal enhancement and complicated cysts or technical artefacts. As > 99% of all diagnostically relevant lesions show pathological enhancement, the first and second subtractions are perfectly suited for the identification of a lesion. Motion artefacts may compromise image assessment and the corresponding non-subtracted pre- and post-cotrast T1w images should always be screened for lesions. False-negative findings at this step are usually associated with a location next to the thoracic wall, in the axillary breast parenchyma, or in the axilla itself. If prior mammography or ultrasound-identified lesions are present, it is important that these be identified as well, using pre-constrast T1w and T2w images, as these lesions may not enhance.

Each lesion description should include:

### Size, localisation, and anatomical relationships

The largest diameter of a lesion should be documented as [cm] in the anterior-posterior direction. In more complex lesions, the diameter should be given in the perpendicular planes. For communication and second-look ultrasound, we prefer a clockwise localisation accompanied by the area in which the lesion is located: retroareolar, central gland, or posterior. This includes a specification of whether breast parenchyma extends into the axilla and whether the lesion is inside or outside the breast parenchyma. The latter strongly hints at an intramammary lymph node. In case of suspected breast cancer, it is also crucial to report the minimal distance toward the nipple, the skin, and the thoracic wall and whether these structures are infiltrated, as these determine the surgical approach.

### Lesion type: Mass, non-mass, or focus?

Enhancing breast lesions present as two phenotypes: mass and non-mass lesions. The category of focus applies only to single spot-like lesions ≤ 5 mm in diameter that are too small to be assessed morphologically. The increase in spatial resolution and the new definition of the revised BI-RADS lexicon to count multiple bilateral foci as BPE have strongly reduced this category assignment in our clinical practice [20]. Note that several asymmetric and grouped foci may rather be described as non-mass lesions. Per definition, mass lesions are clearly space-occupying, whereas these characteristics are not evident in non-mass lesions. Both are associated with different histopathological correlates, as most fibroadenomas, papillomas, and invasive breast cancers present as masses while non-mass lesions include invasive lobular cancers, DCIS, high-risk lesions, and benign proliferative or inflammatory changes. Of note, the Kaiser score is applicable to mass and non-mass lesions alike.

### Diagnostic criteria

Generally, the classifier most suspicious for malignancy applies. If a lesion, e.g., mostly presents a persistent curve, but a small compartment with wash-out not related to artefacts, this more suspicious criterion applies. Similarly, if a generally well-circumscribed lesion shows a single spiculation, this lesion should be classified as spiculated. Considering the limited spatial resolution of breast MRI and the likely presence of artefacts, diagnostic criterion categories should only be applied if unambiguously present: e.g., a possible spiculation or wash-out should not be assigned if not objectively present but rather because of artefacts.

### Margins (Fig. 3)

According to the BI-RADS lexicon, lesion margins can be circumscribed, irregular, or spiculated. Hereby, the probability of malignancy increases from circumscribed to irregular until spiculated.

**Fig. 3:**
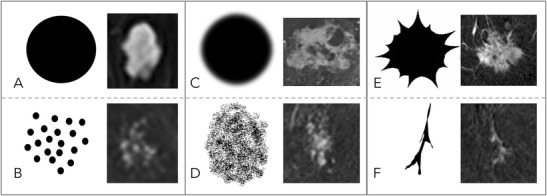
Diagnostic criteria: margins. Margins can be either circumscribed or not circumscribed. Circumscribed margins indicate a benign lesion and are more regularly found in mass (A) than in non-mass (B) lesions. Non-circumscribed margins include irregular (C: mass lesion, D: non-mass lesion), hinting at an intermediate risk of breast cancer, and spiculated. Spiculated margins (E: mass lesions, F: non-mass lesion) are highly suggestive of malignancy. Note that the most suspicious criterion applies; thus, single spiculae in an otherwise circumscribed lesion constitute spiculated margins. This is why this criterion was named the “root sign” by Kaiser

In addition to the standard BI-RADS, we believe that margins should be assessed in both mass and non-mass lesions. Although non-mass lesions will typically not be well circumscribed, spiculated margins can be seen in non-mass lesions. This aspect is illustrated in Fig. 3f.

### Signal-intensity time curve type

The shape of the signal-intensity time curve is determined by initial and delayed enhancement and should be assessed by comparing early or peak and delayed enhancement time points.

1. A continuous signal increase is defined as “persistent”.
2. A steady signal over time is called a “plateau”.
3. A signal drop from the initial to the delayed phase is referred to as “wash-out”.

The probability of malignancy increases from type I to type III curves [17, 19, 21–24]. Persistent indicates benign and wash-out malignant lesions. A plateau is considered an equivocal finding. We recommend visual assessment of curve types, as it is much faster and less biased by motion and/or partial volume artefacts compared to semi-/automatic ROI measurements. Note that all the different methods designed to assess dynamic enhancement curves show comparable results [23, 25–27]. A potential benefit of semi-/automatic ROI measurements has been documented only for non-mass lesions [28].

### Internal enhancement pattern (IEP, Figs. 4, 5)

The Kaiser score dichotomises IEP into suspicious and not suspicious:

**Fig. 4:**
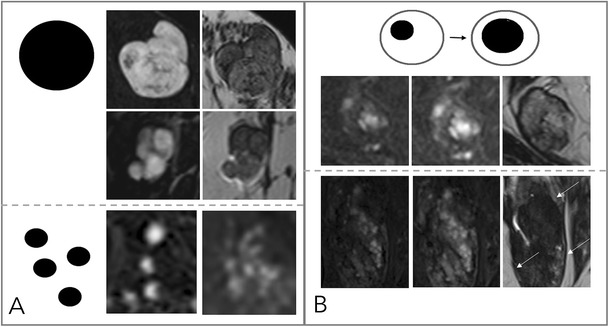
Diagnostic criteria: non-suspicious internal enhancement patterns. A: Homogeneous enhancement suggests benign lesions. Note that even a homogeneous enhancing lesion may show areas of lesser or absent enhancement due to septae and fibrotic parts [A, upper two rows, each early enhanced subtractions (left) and T2w (right)]. Homogeneity is more difficult to assess in non-mass lesions (A, lower row) and includes homogeneous internal morphology. Therefore, this feature was referred to as “stippled” enhancement in the initial BI-RADS lexicon. B: A central or centrifugal enhancement is highly suggestive of a benign lesion. To assess this feature, pre-contrast images need to be considered. While this feature usually applies to mass lesions only (A, upper row, from right to left, early, delayed enhanced, and T2w images), non-mass lesions may also present with this typical benign feature (B, lower row, note the by far larger lesion correlate on the right T2w image, as marked by arrows)

**Fig. 5:**
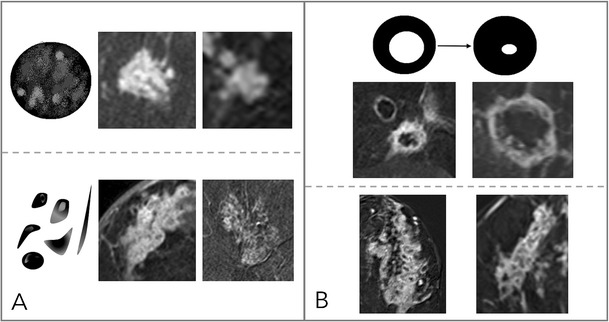
Diagnostic criteria: suspicious internal enhancement patterns. Heterogeneous enhancement may be associated with breast cancer and applies to mass and non-mass lesions (A, upper and lower row, respectively). Specific for malignancy is a centripetal or rim-like enhancement (B). In particular, a broad heterogeneous rim, regularly associated with a delayed enhancement of the central lesion parts, is highly suggestive of breast cancer (B, upper row). However, a thin, rather subtle and homogeneous rim enhancement with absent enhancement of the lesion centre hints at inflammatory conditions, such as cysts, liponecrosis, and abscess (B, upper row, left upper lesion). In non-mass lesions, rim enhancement may appear in a multiple and clustered manner (B, lower row) and is referred to as “clustered ring enhancement” in the BI-RADS lexicon

Non-suspicious IEP is present in case of homogeneous and centrifugal enhancement (Fig. 4).

- Centrifugal enhancement is typical of benign mass lesions, such as fibroadenomas or adenosis. Initially, only a central part of the lesion enhances, slowly followed by the rest of the lesion during the delayed dynamic scans. Especially in larger (> 5–10 mm) lesions, the enhancement may affect only a part of the lesion volume, formerly referred to as central enhancement by the BI-RADS lexicon. The revised BI-RADS omitted this diagnostic feature, which is typical in fibroadenomas because of underuse.
- Homogeneous enhancement is diagnosed if the lesion shows a uniform enhancement without spatial signal variations.

Suspicious IEP is present in case of inhomogeneous and centripetal enhancement (Fig. 5).

- Inhomogeneous enhancement is diagnosed if the lesion shows a non-uniform enhancement in each scan of the dynamic series. Different components are visible within the lesions that show different levels of brightness.
- Centripetal enhancement is highly suggestive of malignant lesions. Initially, the periphery of the lesion enhances, which is why this feature is referred to as “rim” enhancement in the BI-RADS lexicon. Depending on central fibrosis and necrosis, the central part of the lesion shows a delayed or incomplete enhancement. Lack of any centripetal behaviour in rim-enhancing lesions may indicate an inflammatory diagnosis, such as an abscess.

### Oedema (Fig. 6)

Oedema is defined as perifocal, diffuse ipsilateral, or subcutaneous spots of T2w high signal intensity caused by fluid that is not located inside cysts or ectatic ducts. It strongly hints at malignant lesions and suggests lymphangiosis and an increased risk of lymph node metastases [29, 30]. Note that bilateral diffuse oedema is not helpful for the differentiation of lesions, as it is rather associated with cardiac or renal failure. Immediately (hours to days) after biopsy, a false-positive perifocal oedema may occur because of post-interventional changes. If fat saturation is not used, it is important to choose a relatively long TE for T2w sequences (~ 180–200 ms). This will enable reliable differentiation of fat and water because of the higher signal intensity of aqueous substances. Note that STIR and T2w images are equally suited for the assessment of oedema [31].

**Fig. 6:**
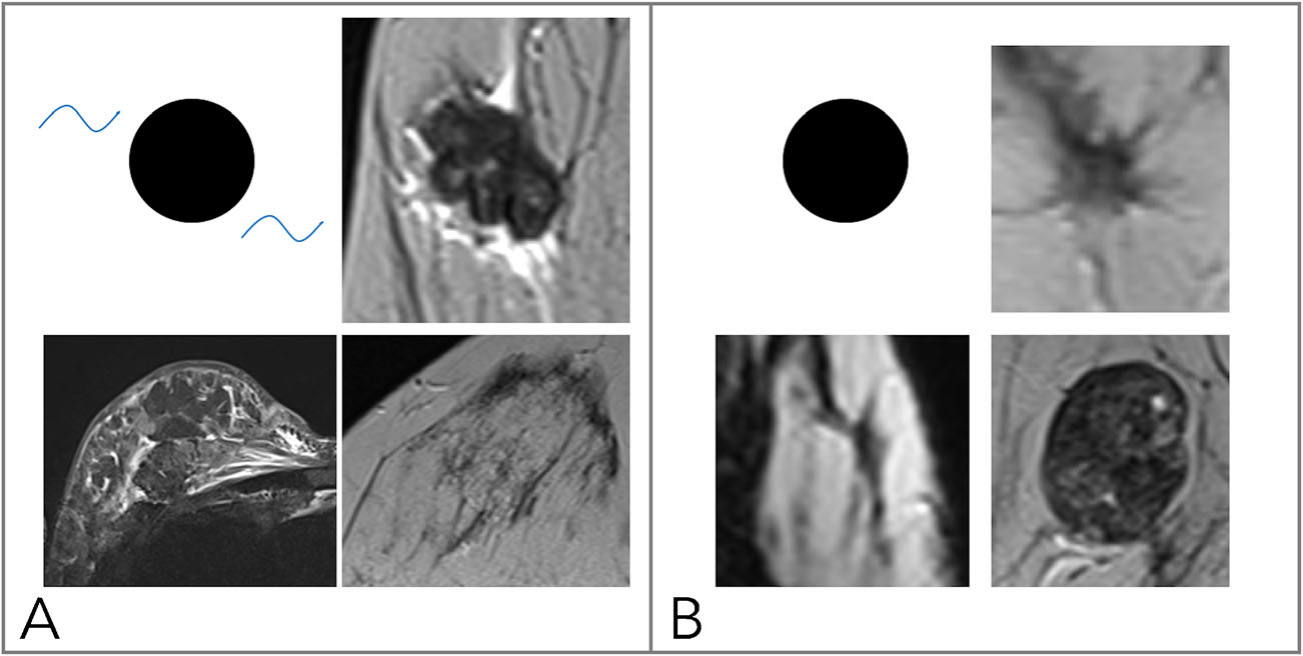
Diagnostic criteria: oedema. As shown in (A), oedema is present if fluid-caused T2w hyperintensity not attributable to cysts or dilated ducts is identified in the breast that harbours an enhancing lesion. Odema may be perifocal [A, upper right (mass) and lower right (non-mass)] or more diffuse (A, lower left, showing subcutaneous, diffuse, and pre-/intrapectoral oedema in an inflammatory breast cancer case with distinct lymphangiosis). B: Absent oedema. The common pitfall causing false-positive oedema assessment is chemical shift artefacts in sharp fat-water interfaces, such as that caused by vessels (e.g., B upper right, lower right) and capsulated fibroadenomas at the parenchyma-fat interface

## The Kaiser score and its translation into BI-RADS category assignments

**Fig. 7:**
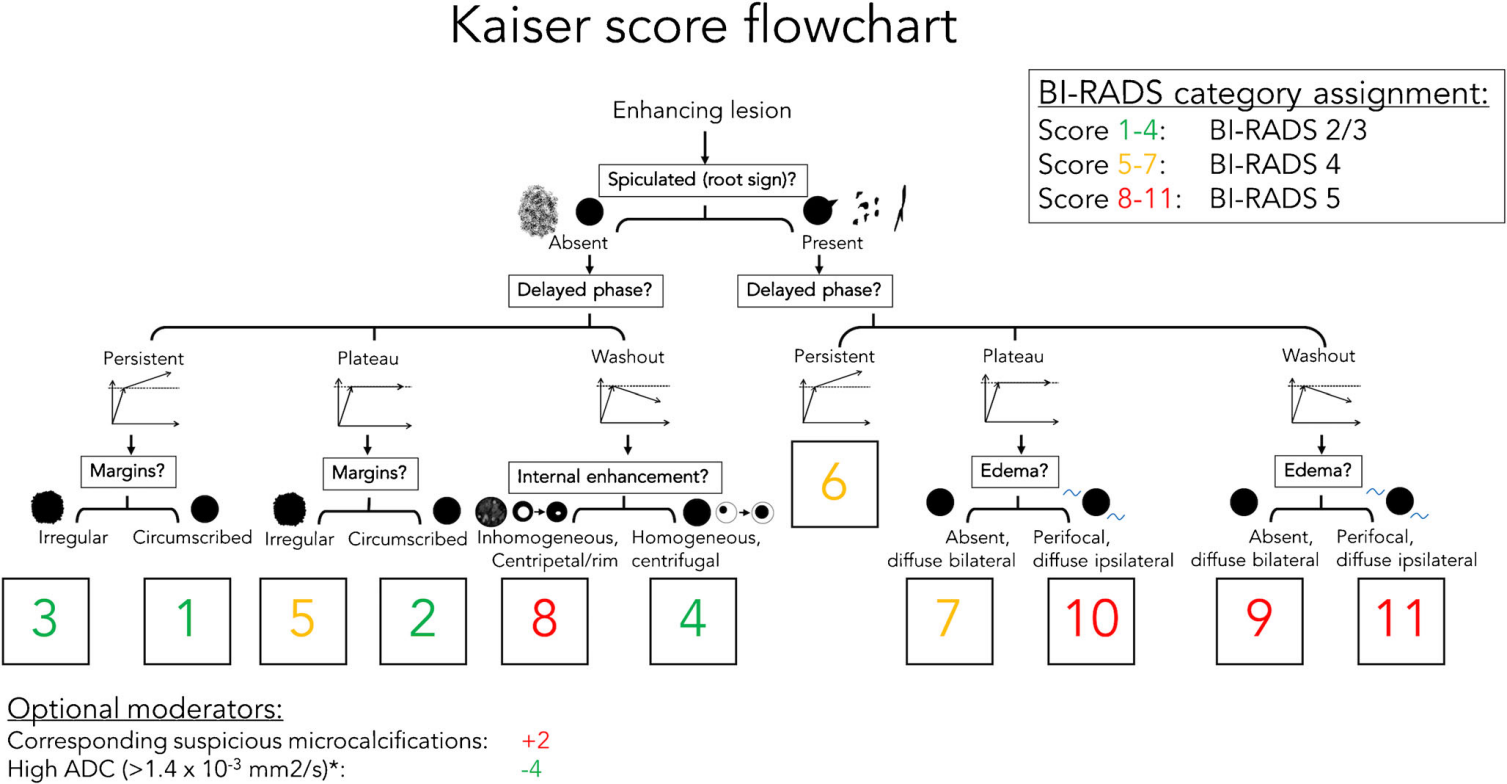
The Kaiser score flowchart. The Kaiser score is assigned by following a simple flowchart from the top to the bottom, which lets the reader assign the presence or absence of four diagnostic criteria (presence of spiculation being a formal subgroup of margin assessment according to BI-RADS). The resulting score is associated with an increasing risk of malignancy (from 1 to 11) and can be translated into BI-RADS categories as follows: 1–4: minimal risk of breast cancer—BI-RADS 2/3; 5–7: intermediate risk of breast cancer—BI-RADS 4; 8–11: high risk of breast cancer—BI-RADS 5. Optional moderators include: presence of suspicious corresponding microcalcifications (+2) and high corresponding ADC values (-4). Further details and explanations are provided in the main text

**Fig. 8:**
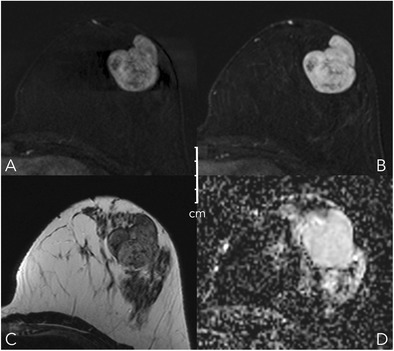
Early (A) and delayed (B) contrast-enhanced subtractions, T2w (C), and ADC map (D). Mass lesion in the left breast of a 27-year-old female, newly palpable after considerable weight loss. The lesion shows no spiculations, a persistent enhancement curve type, and circumscribed margins, corresponding to a Kaiser score of 1 (Fig. 6). Note internal septations and high ADC (2.1*10^-3^ mm^2^/s). This is an unambiguous benign finding, BI-RADS 2. The patient requested a biopsy that revealed a fibroadenoma B2

**Fig. 9:**
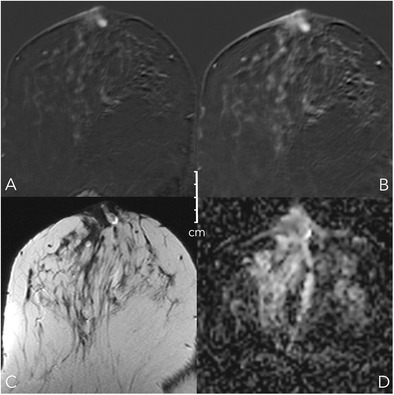
Early (A) and delayed (B) contrast-enhanced subtractions, T2w (C), and ADC map (D). Mass lesion in the left breast of a 41-year-old female with newly developed bloody discharge. The lesion shows no spiculations, circumscribed margins (first subtraction), and a plateau-type enhancement curve, corresponding to a Kaiser score of 2, practically excluding malignancy (Fig. 6). Note the intraductal lesion location on T2w and the intermediate low ADC (1.1*10^-3^ mm^2^/s). Findings are pathognomonic for a benign papilloma. The symptomatic lesion was resected and histopathology revealed a benign papilloma without atypia

**Fig. 10:**
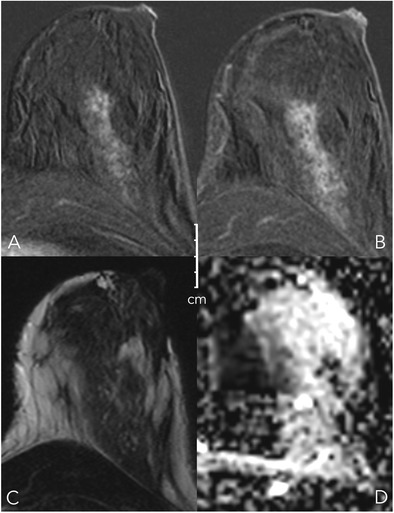
Early (A) and delayed (B) contrast-enhanced subtractions, T2w (C), and ADC map (D). Non-mass lesion in the left breast of a 25-year-old female with pain after stopping breast-feeding and unclear sonographic findings. The lesion shows no spiculations, a persistent enhancement curve type, and irregular margins, corresponding to a Kaiser score of 3 (Fig. 6). Note the internal small cystic correlate that suggests a benign lesion; ADC is unspecific (1.2*10^-3^ mm^2^/s). Together with the clinical symptoms, periductal mastitis must be suspected and it is safe to assign BI-RADS 2. Due to personal preferences, the patient requested a biopsy that revealed chronic periductal mastitis, B2

**Fig. 11:**
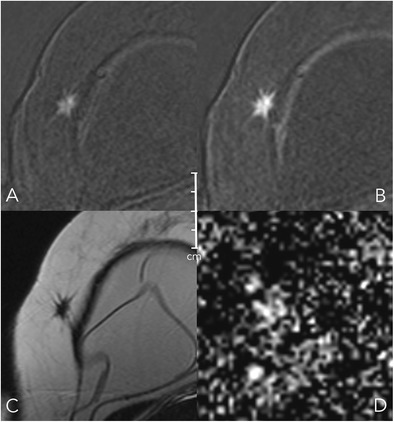
Early (A) and delayed (B) contrast-enhanced subtractions, T2w (C), and ADC map (D). Mass lesion in the right breast of a 50-year-old female undergoing follow-up for breast cancer treatment. The lesion shows spiculations and a persistent enhancement curve type, corresponding to a Kaiser score of 6 (Fig. 6). T2w signal intensity is low (C) and ADC is decreased (0.8*10^-3^ mm^2^/s). Histopathology revealed recurrent invasive ductal cancer G2, B5b

**Fig. 12:**
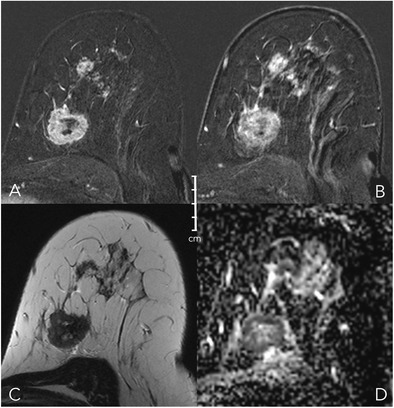
Early (A) and delayed (B) contrast-enhanced subtractions, T2w (C), and ADC map (D). Mass and non-mass lesions in the left breast of a 48-year-old female undergoing MRI for staging purposes because of conventional BI-RADS 5 findings prior to biopsy. The main mass lesion shows multiple subtle spiculations, a wash-out curve type, and a perifocal oedema, corresponding to a Kaiser score of 11 (Fig. 6). Note central necrosis (high signal in C) and low ADC (0.7*10^-3^ mm^2^/s). The anterior lesions show no spiculations, a plateau curve type, and irregular margins, corresponding to a Kaiser score of 5 (Fig. 6). Again, ADC is low (0.9-1*10^-3^ mm^2^/s). Histopathology revealed a multicentric IDC, G3, with a DCIS component, B5b

The Kaiser score (Fig. 7) is a clinical decision rule with which to distinguish benign from malignant breast lesions in bMRI. The Kaiser score is a decision tree organised as a flowchart, which guides a three-step lesion assessment based on the four (presence of spiculation being a formal subgroup of margin assessment according to BI-RADS) independent diagnostic criteria described in the previous section. The result of following the flowchart from the top to the bottom is a diagnostic score that reflects increasing probabilities of malignancy, ranging from 1 to 11. Each score value reflects a specific combination of diagnostic criteria that reflects the lesion phenotypes associated with typical diagnoses (see Table 1 and Figs. 8, 9, 10, 11, 12). This aspect is not only helpful for the differential diagnosis, but also for documentation and teaching purposes. For clinical decision-making, scores below 5 should be considered benign, while biopsy is indicated starting from a score of 5. Obviously, the clinical context needs to be considered: a retroareolar lesion with a Kaiser score of 2 in a female with bloody discharge should be biopsied, as it is likely a benign symptomatic papilloma. Note that scores exceeding 7 indicate a substantial probability of malignancy (BI-RADS 4c/5) and thus require a careful evaluation of consistency between biopsy results and imaging if histopathological work-up reveals a benign diagnosis. According to our clinical experience and empirical research, the Kaiser score can be applied independently from MRI protocols and is helpful for less experienced radiologists. A special feature of the Kaiser score is that it compensates for variabilities in the assessment of single diagnostic criteria. While individual scores may differ between readers, the final diagnostic assessment as suspicious (requiring biopsy) versus not suspicious (not requiring biopsy) is very robust [16].

**Table 1:** Kaiser score categories and their typical histopathological correlates

Kaiser score	Benign	Malignant	Example
1	Fibroadenoma	n.a.	Fig. 8
2	Fibroadenoma, adenosis, papilloma	IDC	Fig. 9
3	Benign epithelial proliferations, inflammatory changes	DCIS	Fig. 10
4	Adenosis	IDC	Fig. 2
5	Benign and atypical epithelial proliferations, papillomatosis, fibroadenoma, fibroadenomatoid hyperplasia	DCIS, IDC	Fig. 12
6	Scar tissue and inflammation	IDC, ILC	Fig. 11
7	Scar tissue and inflammation	IDC, ILC	Fig.
8	Atypical fibroadenoma, adenosis	High proliferative IDC, metastasis, lymphoma	Fig. 13
9	n.a.	IDC, ILC	-
10	n.a.	IDC, ILC	-
11	n.a.	IDC, ILC	Fig. 12

IDC: invasive ductal carcinoma; ILC: invasive lobular carcinoma, n.a.: not applicable

### Moderators of the Kaiser score

**Fig. 13:**
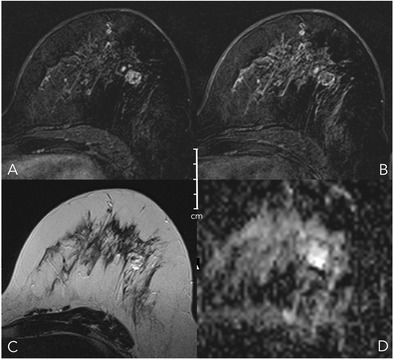
Early (A) and delayed (B) contrast-enhanced subtractions, T2w (C), and ADC map (D). Mass lesion in the left breast of a 50-year-old female with several unclear mass lesions in both breasts; MRI obtained for treatment planning. The lesion shows no spiculations, a wash-out enhancement curve type, and a heterogeneous internal enhancement, corresponding to a Kaiser score of 8 (Fig. 6). Note high signal intensity on T2w. Due to high ADC (1.9*10^-3^ mm^2^/s), the lesion was downgraded to a Kaiser score of 4. Histopathology revealed a fibroadenoma with regressive changes, B2

Generally, BI-RADS category assignments need to be adapted to the specific clinical situation. Newly diagnosed lesions may require a BI-RADS 3 assignment although benign criteria dominate and the Kaiser score is ≤ 4. Developing clinical symptoms, such as bloody discharge or palpable lesions, may fall under this category. In suspicious mammographic microcalcifications, bMRI may be false negative regarding DCIS lesions. In this setting, the pure presence of enhancement may be considered the best diagnostic criterion [7]. Finally, apparent diffusion coefficient (ADC) measurements derived from DWI provide quantitative rule-out criteria for breast cancer. As a result, high ADCs may be used to downgrade Kaiser scores > 4 (see Fig. 13 [32, 33]).

Two recommendations that have not yet been empirically tested with the Kaiser score can be derived from clinical practice and prior reports:

- The Kaiser scores should be upgraded by two points in case of suspicious mammographic microcalcifications to avoid false-negative DCIS diagnoses.
- As a rule of thumb, ADCs exceeding 1.4 *10^-3^ mm^2^/s should be considered an additional criterion for benign lesions [33, 34]. Thus, high ADC values may reduce the Kaiser score by four points. Note that there are malignant lesions that may present with increased ADC values, in particular mucinous cancers [35]. Mucinous cancers are rare findings that usually exhibit typical morphological patterns of malignant lesions. Even if potentially Kaiser scores below the biopsy threshold may be achieved, the clinical situation of a newly diagnosed mass lesion in a postmenopausal patient warrants bioptical verification, rendering the possibility of clinical mismanagement very low. In addition, ADC values of DCIS are higher than those of invasive cancers but still below the ADC threshold defined here [36]. Furthermore, ADC should always be measured in the most vital, enhancing part of the tumour. This is usually in the periphery. Accordingly, even a cancer with extensive central necrosis will not present with elevated ADC values [37]. In lesions presenting as non-mass enhancements (e.g. invasive lobular cancers), special care needs to be taken in measuring the ADC and alternative strategies such as minimum ADC measurements may be helpul [37]. If the ADC map is of low quality or if the lesion cannot be clearly defined, we suggest refraining from taking ADC measurements to avoid false-negative results.

Finally, a management recommendation should be given together with the BI-RADS category assignment. Generally, the simplest imaging method should be used for follow-up or biopsy in case of BI-RADS 3–5 lesions. This is usually second-look ultrasound [38].

## Summary

To achieve high-quality bMRI, patient management, imaging protocols, and set-up must be standardised. Reading of bMRI should be performed in a standardised manner. Lesions should be characterised by the diagnostic criteria, *margins* (circumscribed, irregular, spiculated; the latter also referred to as the root sign), *SI-time curve type, internal enhancement pattern,* and *oedema*. The Kaiser score incorporates these criteria in a diagnostic flowchart that fulfills evidence-based criteria; it can be applied to various bMRI protocols and MRI systems. The Kaiser score can be directly translated into BI-RADS category assignments and provides high diagnostic accuracy with low inter-observer variability. A management recommendation should be an integral part of the report’s conclusion.
